# Molecular characterization and modulated expression of histone acetyltransferases during cold response of the tick *Dermacentor silvarum* (Acari: Ixodidae)

**DOI:** 10.1186/s13071-023-05955-2

**Published:** 2023-10-10

**Authors:** Tingwei Pei, Tianai Zhang, Miao Zhang, Chuks F. Nwanade, Ruotong Wang, Zihao Wang, Ruwei Bai, Zhijun Yu, Jingze Liu

**Affiliations:** 1 Hebei Key Laboratory of Animal Physiology, Biochemistry and Molecular Biology, Hebei Collaborative Innovation Center for Eco-Environment, Hebei Research Center of the Basic Discipline of Cell Biology, Ministry of Education Key Laboratory of Molecular and Cellular Biology, College of Life Sciences, Hebei Normal University, Shijiazhuang, 050024 China; 2 Department of Entomology and Plant Pathology, The University of Tennessee, Knoxville, TN USA

**Keywords:** *Dermacentor silvarum*, Histone acetyltransferase, Cold-stress response, Epigenetic regulation

## Abstract

**Background:**

Histone acetylation is involved in the regulation of stress responses in multiple organisms. *Dermacentor silvarum* is an important vector tick species widely distributed in China, and low temperature is a crucial factor restricting the development of its population. However, knowledge of the histone acetyltransferases and epigenetic mechanisms underlying cold-stress responses in this tick species is limited.

**Methods:**

Histone acetyltransferase genes were characterized in *D. silvarum*, and their relative expressions were determined using qPCR during cold stress. The association and modulation of histone acetyltransferase genes were further explored using RNA interference, and both the H3K9 acetylation level and relative expression of KAT5 protein were evaluated using western blotting.

**Results:**

Three histone acetyltransferase genes were identified and named as *DsCREBBP*, *DsKAT6B*, and *DsKAT5*. Bioinformatics analysis showed that they were unstable hydrophilic proteins, characterized by the conserved structures of CBP (ZnF_TAZ), PHA03247 super family, Creb_binding, and MYST(PLN00104) super family. Fluorescence quantitative PCR showed that the expression of *DsCREBBP*, *DsKAT6B*, and *DsKAT5* increased after 3 days of cold treatment, with subsequent gradual decreases, and was lowest on day 9. Western blotting showed that both the H3K9 acetylation level and relative expression of KAT5 in *D. silvarum* increased after treatment at − 4, 4, and 8 °C for 3 and 6 days, whereas they decreased significantly after a 9-day treatment. RNA interference induced significant gene silencing, and the mortality rate of *D. silvarum* significantly increased at the respective semi-lethal temperatures.

**Conclusion:**

These results imply that histone acetyltransferases play an important role in tick adaptation to low temperatures and lay a foundation for further understanding of the epigenetic regulation of histone acetylation in cold-stressed ticks. Further research is needed to elucidate the mechanisms underlying histone acetylation during cold stress in ticks.

**Graphical Abstract:**

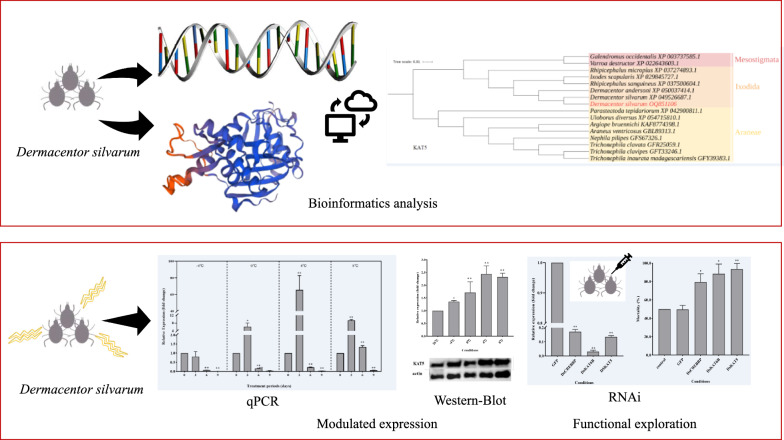

**Supplementary Information:**

The online version contains supplementary material available at 10.1186/s13071-023-05955-2.

## Background

Epigenetics refers to a heritable variation that is independent of changes in the DNA sequence and plays versatile roles in various biological processes, including metamorphosis, development and reproduction, immunity, longevity, and gender-specific phenotypic variation [1]. Among the different epigenetic modifications, histone post-translational modifications (PTMs) are considered a major group of important epigenetic indicators and are closely associated with gene activation or repression [2, 3]. Histone modifications mostly involve acetylation, methylation, phosphorylation, and ubiquitination on lysine or arginine residues in the N-terminus of histones H3 and H4 [4]. As one of the most intensively studied histone modifications, histone acetylation is dynamically modulated by histone acetyltransferase (HAT) and histone deacetylases (HDACs) [5, 6].

HATs are a group of histone-modifying enzymes and are classified into different families based on sequence homology. The HAT families include GCN5-related N-acetyltransferases (GNATs), the MYST proteins that include HBO1 (histone acetyltransferase binding to origin recognition complex), p300/CREB-binding protein (CBP) and p300/CBP-associated factor (PCAF), general transcription factor HATs including the TFIID subunit TBP-associated factor-1 (TAF1), and the nuclear hormone-related HATs SRC1 and ACTR (SRC3) [7, 8]. Most HATs feature a conserved acetyl-CoA-binding core and usually catalyze the transfer of an acetyl group to specific lysine residues [9]. Histone H3 can be acetylated on lysine at sites 4, 9, 14, 18, 23, 27, 36, 56, and 79; histone H4 can be acetylated on lysine at sites 5, 8, 12, 16, 20, and 91; histones H2A and H2B can be acetylated on lysine at sites 5 and 9, and 5, 12, 15, 16, 20, and 120, respectively [10].

Histone acetylation is involved in the regulation of stress responses in multiple organisms [11–13]. In the flesh fly *Sarcophaga bullata*, a reduction in total histone H3 acetylation was found in environmental stress-induced diapausing pupae, and differential expression of HAT genes was observed in pre-diapause, diapause, and post-diapause flies [11]. In the goldenrod gall moth *Epiblema scudderiana*, decreased levels of histone acetyltransferase and histone deacetylase activity were found during cold exposure [14]. In ticks, several histone acetyltransferase genes have been characterized in *Ixodes scapularis* and *Amblyomma maculatum* [15, 16]; however, their roles in response to environmental stress remain unknown.

The tick *Dermacentor silvarum* is mainly distributed in northern China, Russia, and Mongolia [17]. They can transmit many pathogens, including the tick-borne encephalitis virus (TBE), spotted fever group rickettsiae including *Rickettsia raoultii*, *R. slovaca*, and *R. heilongjiangensis*, *Anaplasma phagocytophilum*, *Babesia caballi*, and *Theileria equi*, as well as *Ehrlichia chaffeensis* [18, 19]. Notably, *D. silvarum* can transmit the TBE virus transstadially (from larva to nymph to adult ticks) and transovarially (from adult female tick through eggs) [20]. In North China, *D. silvarum* completes one generation per year in the field and overwinters as a diapausing unfed adult [21]. A previous study indicated that *D. silvarum* is freeze-intolerant, with adults showing more cold hardiness than immature ticks [22]. However, knowledge of histone acetyltransferases and the epigenetic mechanisms underlying the cold-stress response in this tick species is limited.

Therefore, the histone acetyltransferases of *D. silvarum* were identified in the present study, and changes in their relative expression were determined under different low-temperature conditions. The potential functions of histone acetyltransferases under cold stress in *D. silvarum* were further analyzed through RNA interference (RNAi) to shed light on the mechanisms of epigenetic regulation in ticks in response to cold stress, which may contribute to the subsequent control of ticks and tick-borne diseases.

## Methods

### Tick collection and maintenance

Free-living adult ticks of *D. silvarum* were collected from vegetation using flag dragging in the Xiaowutai National Natural Reserve Area (39°50′ to 40°07′ N, 114°47′ to 115°30′ E), Hebei Province, China. They were transferred to the laboratory and maintained in an environmental incubator [temperature 26 ± 1 °C, relative humidity (RH) 75 ± 5%, and 16 h light: 8 h dark]. For feeding, they were placed on the ears of New Zealand white rabbits, as described previously [23]. The second-generation unfed female adults at 2 weeks post-molt were randomly selected for subsequent assays. All the experiments were approved by the Animal Ethics Committee of Hebei Normal University (Protocol Number: IACUC-208102).

### RNA extraction and cDNA synthesis

The unfed female ticks of *D. silvarum* were placed into 1.5-ml Eppendorf (EP) tubes (10 females per group) and sequentially sterilized using ddH_2_O and 75% ethanol via ultrasonic treatment for 15 s. After drying with a sterilized filter paper, they were placed in a pre-cooled mortar and homogenized in liquid nitrogen. The powders were sequentially treated with chloroform, isopropanol, and ethanol to extract total RNA [24]. The integrity and quality of the RNA were evaluated using a NanoDrop ND-2000 spectrophotometer (Thermo Fisher Scientific, Waltham, MA, USA), with a ratio of A260/A280 typically > 2.0, followed by 1% agarose gel electrophoresis. TransScript^®^ One-Step gDNA Removal and cDNA Synthesis SuperMix (TransGen Biotech Co., Ltd, Beijing, China) were used to synthesize cDNA according to the manufacturer’s protocol. A mixture of 1–7 µl total RNA, 1 µl Anchored Oligo (dT) 18 Primer (0.5 μg/µl), 0–6 µl RNase-free water, 10 µl 2 × TS Reaction Mix, 1 µl RI Enzyme Mix, and 1 µl gDNA Remover were used for PCR. The cDNA products were obtained under the conditions of 42 °C for 30 min, followed by 85 °C for 5 s.

Premier version 5.0 for Windows (PREMIER Biosoft International, Palo Alto, CA, USA) was used to design the primers (Table 1), and amplification of the target genes was carried out under the following conditions: initial 2 min denaturation at 94 °C, 40 cycles of 30 s at 94 °C, 30 s at the melting temperature Tm (60 °C) of the gene-specific primers, and 30 s at 72 °C, followed by a final extension at 72 °C for 10 min, on an Applied Biosystems Veriti 96-Well Thermal Cycler (Life Technologies Ltd., Marsiling, Singapore). The amplified fragments were verified and separated on a 1% agarose gel. Bands of the expected sizes were excised and purified using an EG101-01 EasyPure Quick Gel Extraction Kit (TransGen) according to the manufacturer’s protocol. The purified products were sequenced and used for subsequent analyses.

**Table 1:** Primers for the histone acetyltransferase genes of *D. silvarum*

Genes	Primers (5ʹ-3ʹ)
*DsCREBBP*	F_1_: GACCCCAAGCAGAACAACC
	R_1_: GTAAGGCGGGCGTCATTT
	R_1_: GTAAGGCGGGCGTCATTT
	F_2_: AAATGACGCCCGCCTTAC
	R_2_: GTCCCGACGATAATCTTCAGG
	F_3_: ATCGCCACCAGTTCATAAGG
	R_3_: CCCATCACCTCTGTAGTCCTG
*DsKAT6B*	F_1_: CAGCGAAGACACTCAGGGT
	R_1_: CGATAACTACAAAGACAGCC
	F_2_: GCTGATGACTATGCGGCT
	R_2_: CTATGTGCTCACCAAGAATG
	F_3_: ATCATTCTTGGTGAGCACATAG
	R_3_: CCATCAGATTTGCCTCCAT
	F_4_: CCAAACTCAATAGCAGCG
	R_4_: AAGAAAGAGGGGGAACACT
	F_5_: AAACTCAATAGCAGCGGG
	R_5_: ACCATCAGATTTGCCTCC
*DsKAT5*	F_1_: GCATCGCTACAAAATCGC
	R_1_: TGACACCAAAACCCTCTCT
	F_2_: CTTTTCAAGACAGAGAGGGTTT
	R_2_: GCCCTCGTCCACCATCA
	F_3_: GATGGTGGACGAGGGCA
	R_3_: TCTTGACACTGCCACCTTACC
	F_4_: TTGCCTTCAAACTTGGACAG
	R_4_: CTTCTCGCCATACCCACA
	F_5_: CTGTGGGTATGGCGAGAAGT
	R_5_: GAGACTTTTGCGGGTTGGA

### Bioinformatic analysis

DNAMAN (Lynnon Biosoft, San Ramon, CA, USA) and BLASTn (http://www.ncbi.nlm.nih.gov/BLAST) were used for sequence alignment and identity analyses. Phylogenetic relationships were constructed using the BioEdit (http://www.mbio.ncsu.edu/BioEdit/BioEdit.html) and MEGA11 software, and the evolutionary tree was annotated in the iTOL webtool (https://itol.embl.de/). The NCBI Conserved Domain Search (CD-Search; https://www.ncbi.nlm.nih.gov/Structure/cdd/cdd.shtml) service was used to search for conserved domains, which were subsequently constructed using Illustrator of Biological Sequence (IBS) v1.0. The physicochemical properties of the corresponding proteins were predicted through Expasy (http://www.expasy.org), the DiANNA website (http://bioinformatics.bc.edu/clotelab/DiANNA/), and BioEdit software, whereas the structures of the histone acetyltransferases were predicted using the SOPMA (https://npsa-prabi.ibcp.fr/cgi-bin/npsa_automat.pl?page=/NPSA/npsa_sopma.html) and Swiss-Model online websites (https://swissmodel.expasy.org/).

### Relative expression of histone acetyltransferase genes of *D. silvarum* under cold treatment

For cold treatment, 36 groups of ticks of *D. silvarum* (10 unfed females per group) were used and treated under different low temperatures (− 4, 0, 4, and 8 °C) for 3, 6, and 9 days, respectively, with evaluations at each temperature repeated three times. Ticks maintained at 26 °C in the environmental incubator served as the control. Quantitative (real-time) PCR (qRT-PCR) was used to determine the relative expression of histone acetyltransferase genes under different cold treatments, with actin serving as the reference gene. Briefly, 20 μl standard PCR reaction mixture was amplified with 1 μl of the above synthesized cDNA, 0.4 µl gene-specific primers (forward and reverse), and 10 µl 2 × TransStart^®^ Top Green qPCR SuperMix (TransGen). The conditions were set as follows: initial 30 s denaturation at 94 °C, 40 cycles of 5 s at 94 °C, 30 s at 60 °C, and 1 min at 95 °C, followed by a final extension at 55 °C for 30 s. The fold change of gene expression levels was calculated using 2^−ΔΔCt^, and figures were prepared using the GraphPad Prism 8.0 software (USA).

### Western blotting

After cold treatment, groups of ticks (10 unfed females per group) were sterilized and ground in liquid nitrogen; the powders were transferred to a pre-cooled 1.5-ml EP tube containing 200 μl RIPA lysis buffer. After vortexing and centrifugation at 13,000 rpm for 10 min at 4 °C, the supernatant was transferred to a new pre-cooled 1.5-ml EP tube. A bicinchoninic acid (BCA) Protein Assay Kit (CWBIO, Jiangsu, China) was used to determine protein concentration, which was diluted with sterile phosphate-buffered saline (PBS; pH 7.4) to a final concentration of 2.2 μg/μl. Subsequently, samples were dissolved in equal amounts of 0.1 M Tris–HCl (pH 6.8) containing 2% SDS, 5% 2-mercaptoethanol, 10% glycerol, and 0.05% bromophenol blue, followed by 2 min boiling. The SDS-PAGE was performed on 12% separating gels with 4% stacking gels containing 0.1% SDS using a PAGE Gel Preparation Kit (Epizyme, USA). Electrophoresis was performed at a voltage of 80 V for the stacking gel and at 120 V for the separation gel. Subsequently, electrotransfer onto polyvinylidene difluoride (PVDF) membranes was carried out at 22 V for 30 min using a Trans-Blot SD apparatus (Bio-Rad, CA, USA). After blocking and washing, the membrane was incubated overnight with a monoclonal KAT5 antibody (1:2000) or H3K9 antibody (1:500) (GeneTex, TX, USA). After three washes with 1 × TBST (Tris-buffered saline with 0.1% Tween R 20 detergent) for 15 min, the membrane was incubated with horseradish peroxidase (HRP)-conjugated goat anti-mouse IgG for 2 h at room temperature. The signal was detected through an ultra-sensitive enhanced chemiluminescent (ECL) substrate using a SuperSignal^™^ West Femto Trial Kit (Thermo Scientific), visualized, and analyzed using the Image Lab Software (Bio-Rad).

### RNAi

For RNAi, the T7 promoter sequence (5ʹ TAATACGACTCACTATAGG 3ʹ) was added to the 5′-end of each primer and used for dsRNA synthesis; the final concentration of dsRNA was adjusted to 8000 ng/µl. Before injection, each group of ticks (10 unfed females) was sterilized sequentially using ddH_2_O, hydrogen peroxide, and ddH_2_O and immobilized on the dorsal side up using double-sided sticky tape. Microinjections were carried out using 10-μl microliter syringes (Hamilton, Nevada, USA) through the third and fourth coxa, with 2 µl dsRNA for each tick. The control group was injected with dsRNA-GFP. Subsequently, the ticks were allowed to recover for 24 h in the environmental incubator (26 ± 1 °C, RH 75 ± 5%, and 16 h light:8 h dark). Target gene silencing efficiency was then evaluated using qRT-PCR, as described above.

After confirmation of target gene silencing, the *D. silvarum* ticks were exposed to a lower-lethal temperature of – 22 °C for 2 h [22] before mortality rates were calculated. Ticks were considered dead if they could not coordinate their appendages after stimulation with CO_2_.

## Results

### Molecular characterization of histone acetyltransferase genes of *D. silvarum*

**Fig. 1:**
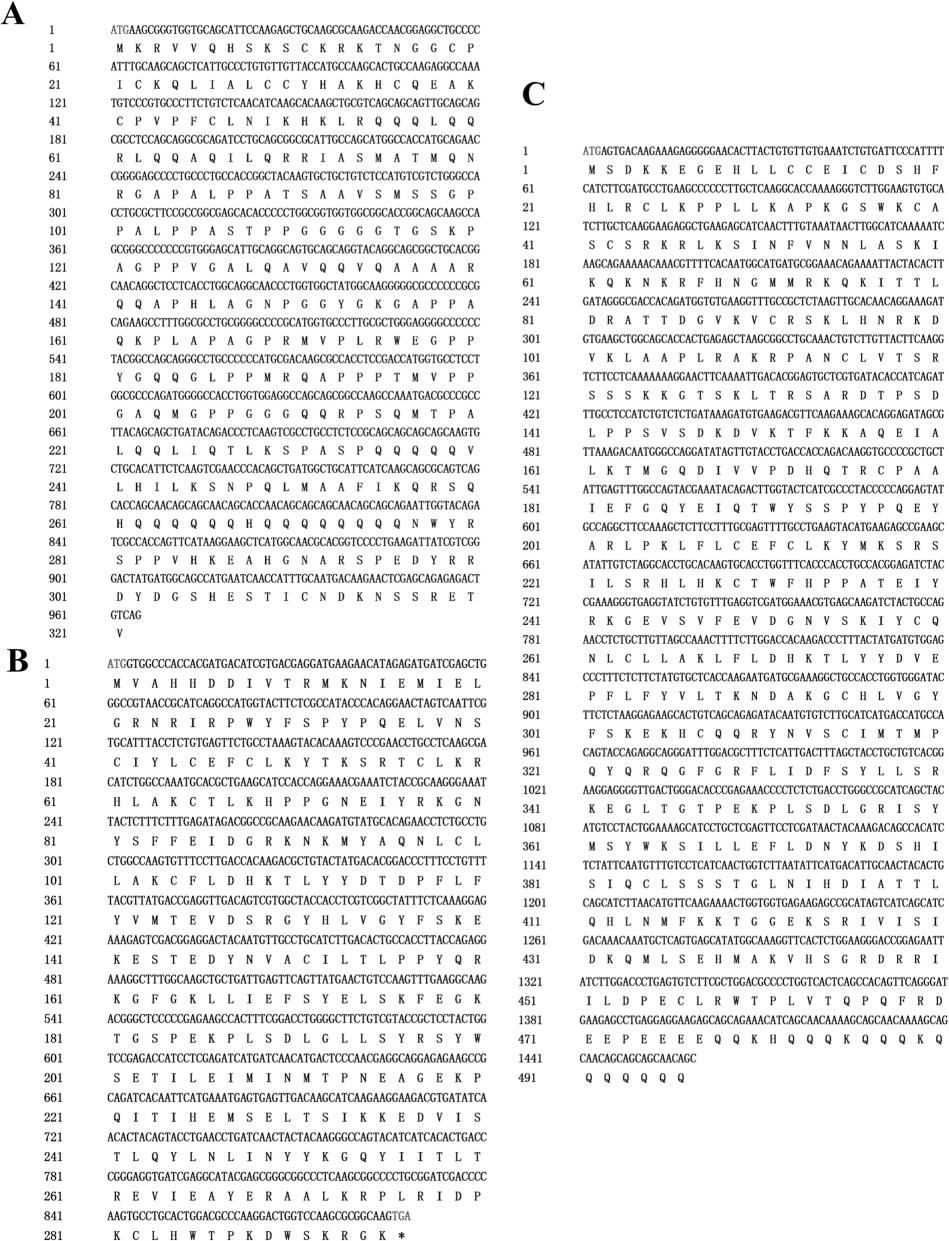
Nucleotide and encoded amino acid sequences of histone acetyltransferases of *Dermacentor silvarum* (**A** *DsCREBBP*; **B** *DsKAT5*; **C** *DsKAT6B*)

**Fig. 2:**
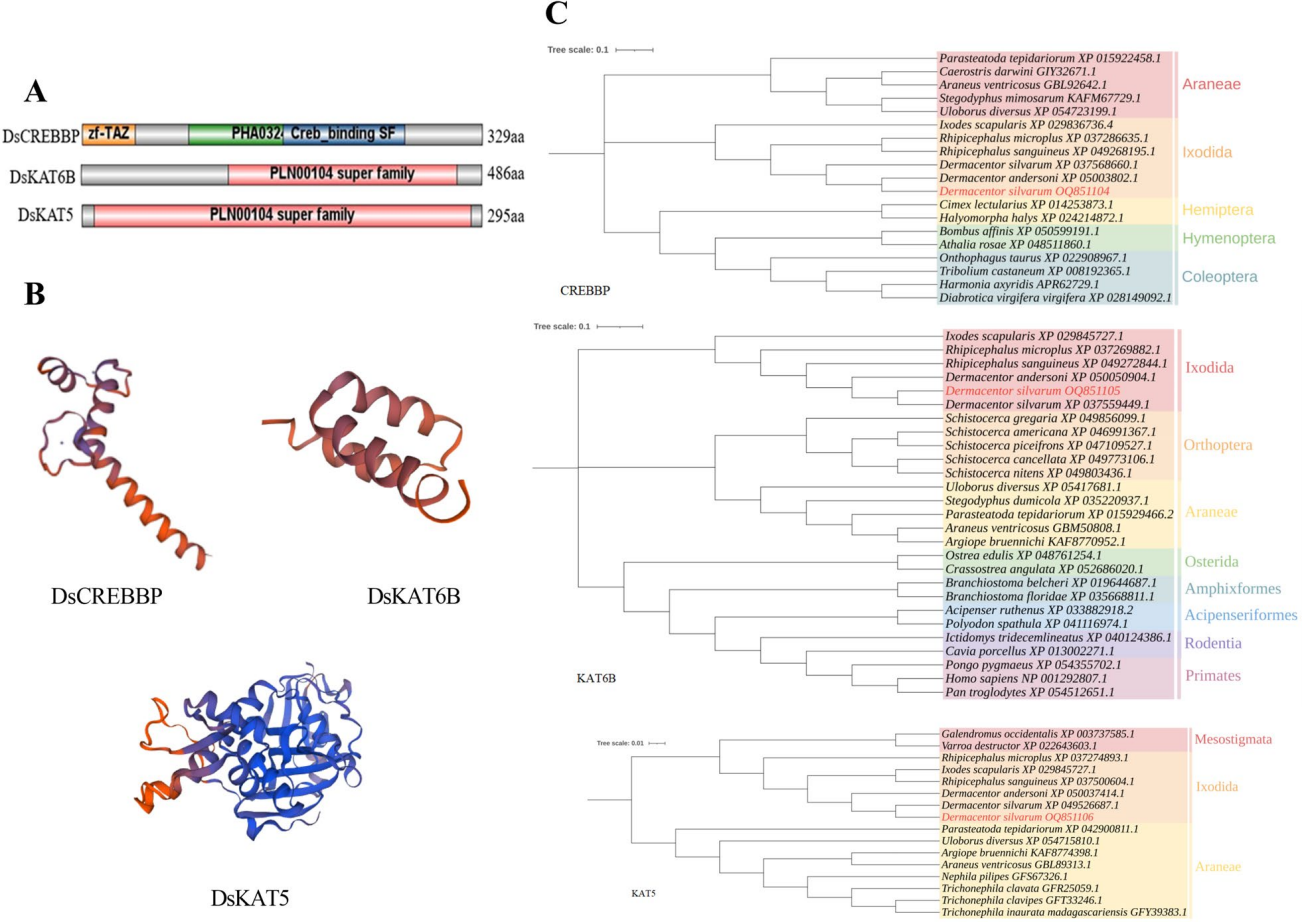
Bioinformatic analysis of the histone acetyltransferases in *Dermacentor silvarum.* **A** schematic map of the conserved domain; **B** predicted tertiary structures using the Swiss-Model online website; **C** phylogenetic tree of the amino acid sequences

Three histone acetyltransferase genes, with lengths of 1106, 1525, and 1848 bp, were cloned from *D. silvarum* ticks, and they were named *DsCREBBP*, *DsKAT6B*, and *DsKAT5*, respectively (Fig. 1). The sequences were deposited in NCBI under the accession numbers QQ851104, QQ851105, and QQ851106, respectively. They displayed high similarity to the histone acetyltransferase genes of *Dermacentor andersoni*, *Rhipicephalus microplus*, and *R. sanguineus* (Additional file 1: Fig. S1). The molecular weight of DsCREBBP, DsKAT6B, and DsKAT5 was 34.64 kDa, 56.35 kDa, and 34.80 kDa, respectively (Table 2). The NCBI-Conserved Domains prediction showed that DsCREBBP contains zf-TAZ and TAZ zinc finger domains and features PHA03247 and Creb_binding domains; DsKAT6B and DsKAT5 feature the MYST (PLN00104) domain (Fig. 2A). The 3D structure prediction was carried out using Swiss-Model, and the Global Model Quality Estimation (GMQE) values of DsCREBBP, DsKAT6B, and DsKAT5 reached 0.11, 0.14, and 0.81, respectively (Fig. 2B). Phylogenetic analysis showed that DsCREBBP, DsKAT6B, and DsKAT5 closely clustered with that of the tick *D. andersoni* and formed one clade with sequences from other Ixodidae tick species, which were clearly separated from those of Araneae, Orthoptera, and Hemiptera (Fig. 2C).

**Table 2:** Physicochemical property of the histone acetyltransferase proteins of *Dermacentor silvarum*

Physicochemical property	Proteins		
	DsCREBBP	DsKAT6B	DsKAT5
Total number of atoms	4829	7927	4896
Molecular weight (kDa)	34.64	56.35	34.80
Theoretical PI	10.28	9.48	8.77
Hydrophobicity index	− 0.831	− 0.654	− 0.482
Instability index	85.16	57.35	48.19
Aliphatic index	56.67	73.62	81.97

The molecular formulas of DsCREBBP, DsKAT6B, and DsKAT5 were C_1491_H_2403_N_477_O_439_S_19_, C_2493_H_3975_N_713_O_716_S_30_, and C_1579_H_2448_N_408_O_444_S_17_, respectively, indicating unstable hydrophilic proteins (Additional file 2: Fig. S2). Signal peptides or trans-membrane domains were not detected in DsCREBBP, DsKAT6B, or DsKAT5. DsCREBBP and DsKAT5 were mainly localized in the nucleus, whereas DsKAT6B was mainly localized in the mitochondria, with a probability of 30.4% (Table 3). The three histone acetyltransferases belong to a mixed secondary structure, with alpha helix and random coil accounting for a large proportion, whereas the proportion of beta turns is relatively low (Table 4).

**Table 3:** Subcellular localization of histone acetyltransferase proteins in *Dermacentor silvarum*

Subcellular localization	DsCREBBP (%)	DsKAT6B (%)	DsKAT5 (%)
Nucleus	56.5	17.4	87.0
Mitochondrion	39.1	30.4	8.7
Cytoplasm	4.3	17.4	
Secretory vesicles		8.7	
Extracellula		8.7	
Endoplasmic reticulum		13.0	
Peroxisome		4.3	4.3

**Table 4:** Secondary structure of histone acetyltransferase proteins in *Dermacentor silvarum*

HAT	Alpha helix (Hh) (%)	Extended strand (Ee) (%)	Beta turn (Tt) (%)	Random coil (Cc) (%)
DsCREBBP	25.86	8.41	5.92	59.81
DsKAT6B	32.3	14.81	4.32	48.56
DsKAT5	36.27	21.69	5.42	36.61

### Relative expression of histone acetyltransferase genes of *D. silvarum* under cold treatment

**Fig. 3:**
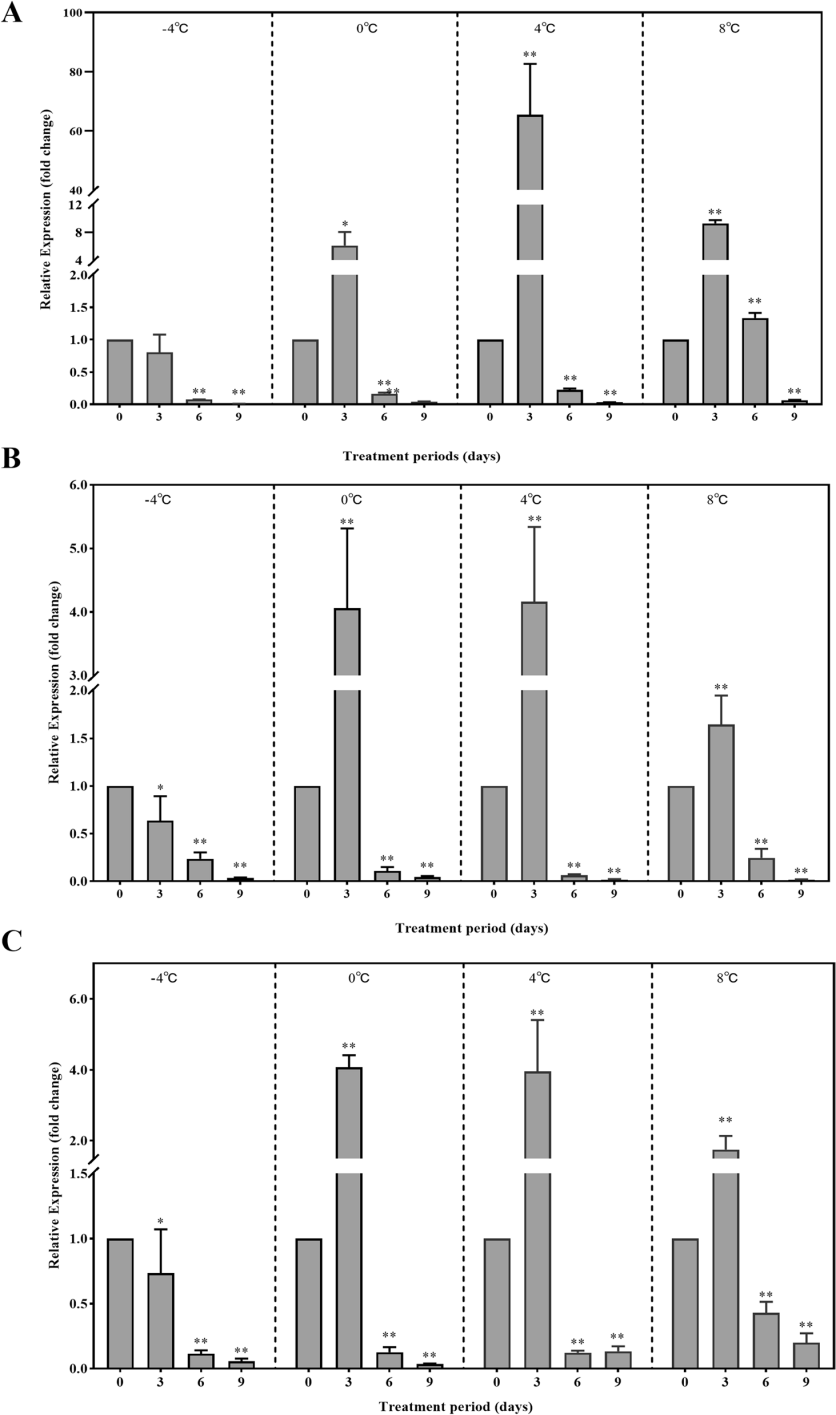
Expression of *DsCREBBP, DsKAT6B*, and *DsKAT5* of *Dermacentor silvarum* exposed to different cold treatment conditions. **A** *DsCREBBP;* **B** *DsKAT6B*; **C** *DsKAT5*. Compared to the control group, * indicates a statistical difference using unpaired t-test at *P* < 0.05 and ** at *P* < 0.01

*DsCREBBP*, *DsKAT6B*, and *DsKAT5* were expressed throughout the entire cold-treatment duration, although their expression levels varied under different temperatures and periods. Under 0, 4, and 8 °C, *DsCREBBP* expression increased within 3 days and reached its highest point on day 3 (*P* < 0.05 or *P* < 0.01), followed by a downward trend (*P* < 0.05). By day 9, the decrease in expression was highly significant (*P* < 0.01). Under the – 4 °C treatment, *DsCREBBP* expression remained at a decreasing trend within 6 days (*P* < 0.01), whereas the decrease was highly significant by day 9 (*P* < 0.01) (Fig. 3A). Under treatment at 0, 4 and 8 °C, *DsKAT6B* expression showed an upward trend within 3 days, reached its highest point on day 3 (*P* < 0.01), and then decreased significantly by day 9 (*P* < 0.01). Under the − 4 °C conditions, moderate decrease was observed within the first 3 days, whereas a highly significant decrease in its expression was noted by day 9 (*P* < 0.01) (Fig. 3B). Under the 0, 4, and 8 °C treatment, *DsKAT5* expression increased within 3 days, reaching its highest point on day 3 (*P* < 0.01), followed by a highly significant decrease on day 9 (*P* < 0.01). Under the − 4 °C conditions, a decrease was recorded within 3 days, whereas a downward trend was subsequently noted (*P* < 0.01). By day 9, a highly significant decrease in *DsKAT5* expression was recorded (*P* < 0.01) (Fig. 3C).

### Modification of H3K9 and relative expression of KAT5

**Fig. 4:**
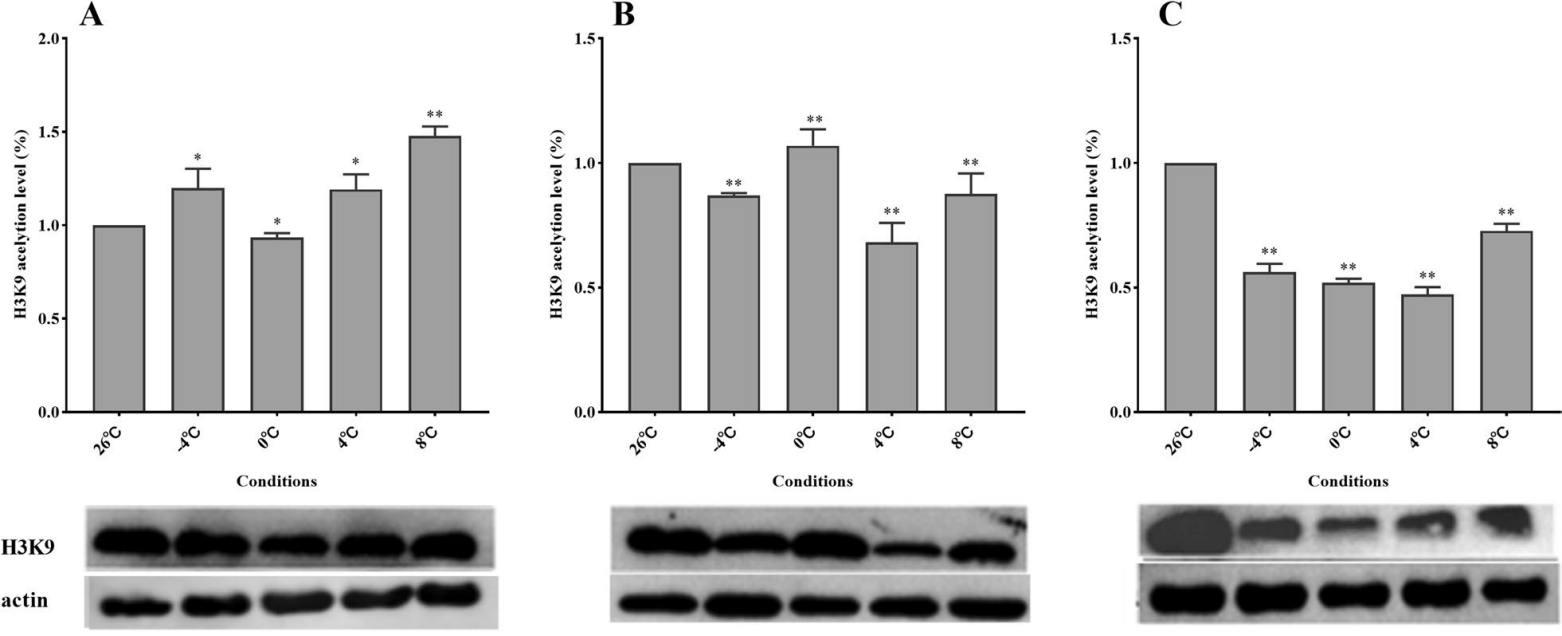
Levels of H3K9 acetylation in *Dermacentor silvarum* under different cold treatment conditions. **A** cold treatment for 3 days; **B** cold treatment for 6 days; **C** cold treatment for 9 days. Analyses were performed with three replications per treatment. Compared to the 26 °C group, * indicates a statistical difference using unpaired t-test at *P* < 0.05 and ** at *P* < 0.01

**Fig. 5:**
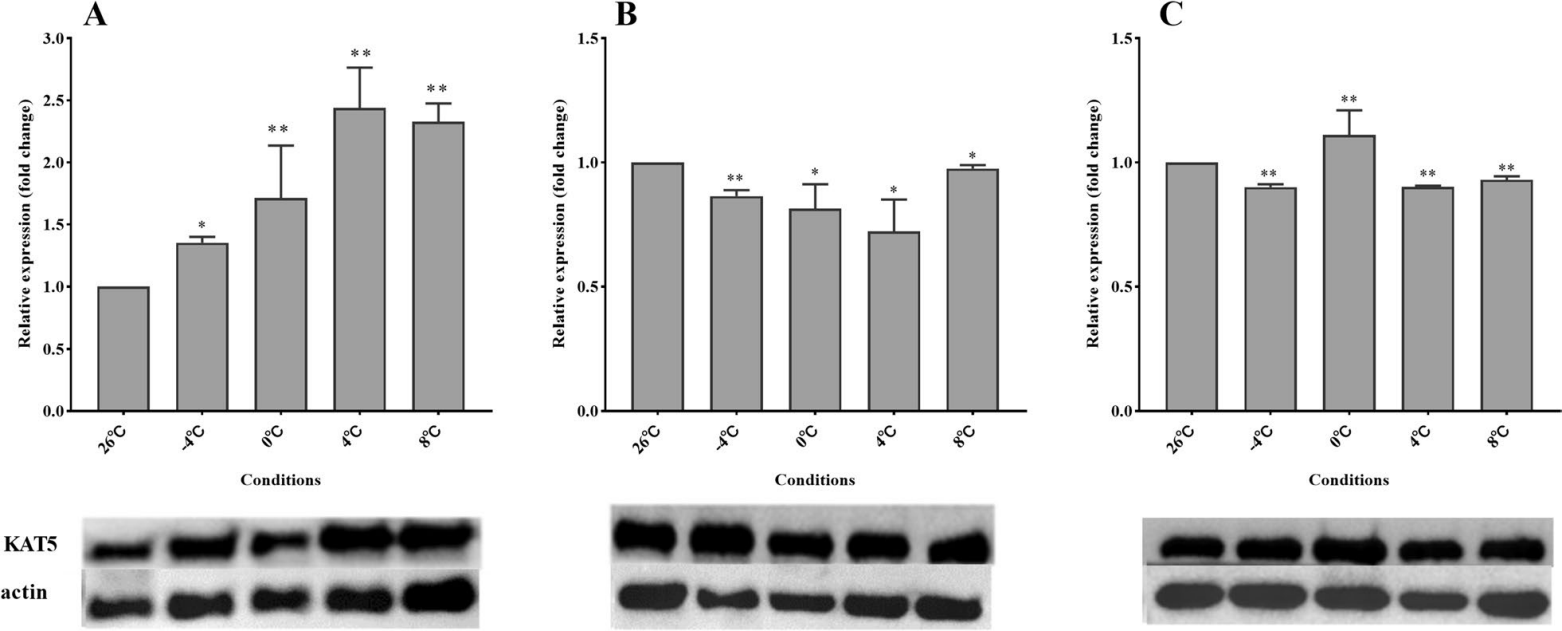
Relative protein expression of KAT5 in *Dermacentor silvarum* under different cold treatment conditions. **A**, **B**, and **C**: cold treatment for 3, 6, and 9 days, respectively. Analyses were performed with three replications per treatment. Compared to the 26 °C group, * indicates a statistical difference using unpaired t-test at *P* < 0.05 and ** at *P* < 0.01

The H3K9 acetylation level in *D. silvarum* changed significantly after cold treatment (*P* < 0.05). After 3 days of cold treatment, the H3K9 acetylation level decreased at 0 °C, whereas it increased at − 4, 4, and 8 °C treatments. The H3K9 acetylation level continued to decrease with extended treatment duration (Fig. 4, Additional file 3: Fig. S3). The protein expression of KAT5 in *D. silvarum* changed significantly (*P* < 0.05) on day 3 of treatment. After 3 days of cold treatment, KAT5 expression was upregulated, whereas with the extension of cold treatment periods, its expression decreased (Fig. 5, Additional file 4: Fig. S4).

### Effect of RNAi on gene expression

**Fig. 6:**
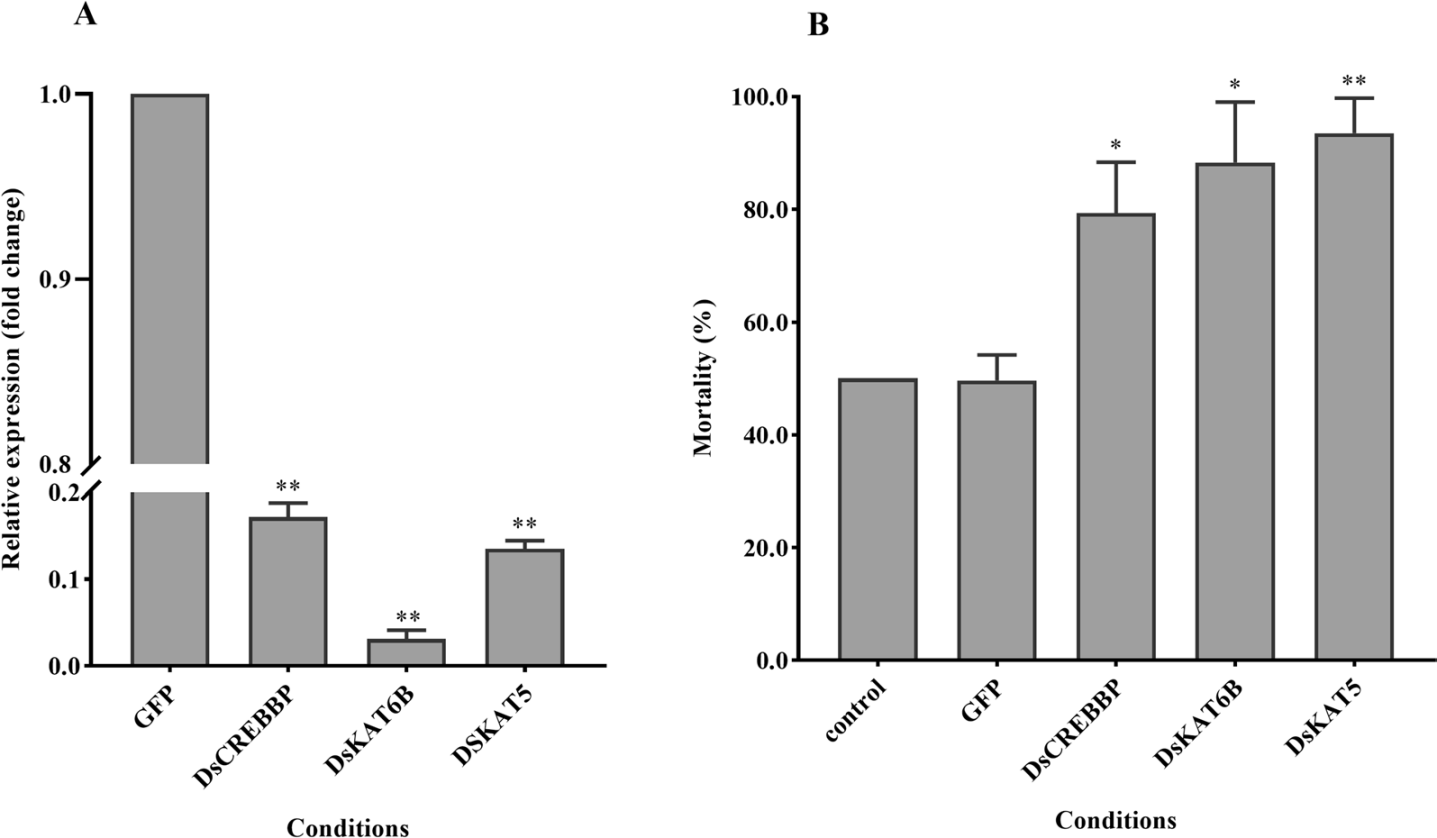
Effects of RNA interference (RNAi) on cold tolerance in *Dermacentor silvarum.* **A** Relative expression of histone acetyltransferase genes after RNAi; **B** mortality of *D. silvarum* under a lower lethal temperature treatment after RNAi. Compared to the control group, * indicates a statistical difference using unpaired t-test at *P* < 0.05 and ** at *P* < 0.01

The expression of the histone acetyltransferase genes was significantly reduced by RNAi (*P* < 0.01), with the average silencing efficiency for *DsCREBBP*, *DsKAT6B*, and *DsKAT5* reaching 84, 98, and 89%, respectively (Fig. 6A). After RNAi, *D. silvarum* ticks were exposed to the lower lethal temperature (LT50) (− 22 °C) for 2 h, and the mortality rates were recorded. The results showed that after injection of *DsCREBBP* or *DsKAT6B*, *D. silvarum* mortality increased significantly compared with that after injections of GFP dsRNA (*P* < 0.05). After *DsKAT5* injection, a highly significant increase in *D. silvarum* mortality was observed (*P* < 0.01) (Fig. 6B).

## Discussion

Histone acetylation plays an important role in the regulation of gene transcription by modifying chromatins, which are highly dynamic in response to environmental stress [25]. In the present study, three histone acetyltransferase genes, *DsCREBBP*, *DsKAT6B*, and *DsKAT5*, were identified in *D. silvarum*, and their association with the cold-stress response of *D. silvarum* was explored.

The CREB-binding protein (CBP, also called CREBBP or KAT3A) and its vertebrate paralog p300 (also called EP300 or KAT3B) are prominent global epigenetic and transcriptional regulators that serve as coactivators of many transcription factors and regulate their expressions by acetylating histones [26, 27]. P300/CBP is highly conserved, with TAZ domains (TAZ1 and TAZ2) that interact with a variety of transcriptional activators, which participate in multiple physiological events, including growth, development, and plasticity of many multicellular organisms [28–30]. Similarly, the *DsCREBBP* characterized in *D. silvarum* featured zf-TAZ and TAZ zinc finger domains as well as PHA03247 and Creb_binding domains. The MYST domain was detected in *D. silvarum DsKAT6B* and *DsKAT5*, indicating that these belong to the MYST family of acetyltransferases. The identified five members of the MYST family of acetyltransferases comprise KAT5, KAT6A (formerly known as MOZ and MYST3), KAT6B (formerly known as MORF and MYST4), KAT7, and KAT8 [25]. The MYST domain can bind to DNA through the zinc finger and helix-turn-helix motifs and can enhance acetylation [31]. In addition, DsCREBBP, DsKAT6B, and DsKAT5 from *D. silvarum* were all unstable hydrophilic proteins, indicating relatively high thermal stability. All proteins were non-secretory and did not contain transmembrane regions. The subcellular localization results showed that most proteins were distributed in the nucleus, which confirmed that they may play an important role in the histones of chromosomes in the nucleus [32].

As a histone acetyltransferase, p300/CBP plays diverse roles in insects. For example, p300/CBP is a crucial factor in the reproduction, embryogenesis, and longevity of the pea aphid *Acyrthosiphon pisum* [30] and can also cause embryonic defects [33] and developmental abnormalities [34] in *Drosophila melanogaster*. In the present study, the overall expression levels of *DsCREBBP* showed a decreasing trend after cold treatment in *D. silvarum*. Similarly, the relative expression of *DsKAT6B* and *DsKAT5* of *D. silvarum* decreased after cold treatment. In *E. scudderiana*, decreases in total HAT activity were observed under treatment at 5 and − 15 °C [14]. This may be attributed to the hypometabolic and global gene-silencing state in many freeze-avoidance organisms during cold stress [35].

H3K9 acetylation levels in *D. silvarum* were found to decrease under cold treatment for 6 and 9 days, and this decreasing trend was consistent with the relative expression of *DsCREBBP* under cold treatment. Similar results were reported for *Epiblema strenuana*, in which significant downregulation of H3K9, H3K18, and H4K8 acetylation levels was found during exposure to 15 °C [14]. In addition, a 40–60% decrease in acetylated H3 content was detected in *T. scripta elegans* soaked in cold water during winter [36]. A decrease in histone H3 acetylation levels has also been detected in some insects in diapause [37]. Changes in the acetylation levels of histone H3 have been observed during the three stages of inducing, maintaining, and relieving diapause in the pupae of *Sarcophaga crassipalpis*, and the occurrence of diapause in the pupae is mainly influenced by the expression of low-acetylated histone H3 [11].

The expression of DsKAT5 significantly decreased in *D. silvarum* after cold treatment for 6 and 9 days, which is consistent with the relative expression of *DsKAT5* under cold treatment. Similar results were found in *E. strenuana*, which showed significantly decreased expression levels of the MYST2 protein after exposure to 15 °C for 4 h [14]. Histone modification is dynamic and changes occur rapidly, which may result in differences in acetyltransferase and acetylation levels. After injection of dsRNA of *DsCREBBP*, *DsKAT6B*, and *DsKAT5*, increased mortality rates of *D. silvarum* were observed, indicating that they may play an important role in the cold-stress response of *D. silvarum*. However, the epigenetic mechanism underlying cold adaptation in ticks requires further exploration.

## Conclusion

The present study characterized three histone acetyltransferase genes in *D. silvarum*, named *DsCREBBP*, *DsKAT6B*, and *DsKAT5*, which showed a positive association with cold stress in *D. silvarum*. Additionally, the H3K9 acetylation levels in *D. silvarum* were consistent with the relative expression of *DsCREBBP*. After injections of dsRNA of *DsCREBBP*, *DsKAT6B*, and *DsKAT5*, *D. silvarum* mortality increased, indicating that histone acetyltransferases may play an important role in the cold-stress response in this species. However, further research is needed to elucidate the mechanisms underlying histone acetylation in cold-stressed ticks.

### Supplementary Information

**Additional file 1**: **Fig. S1.** Homology comparison of the histone acetyltransferases in *Dermacentor silvarum* (**A**: *DsCREBBP*; **B**: *DsKAT6B*; **C**: *DsKAT5*).

**Additional file 2: Fig. S2.** Prediction of hydrophobicity of histone acetyltransferases in *Dermacentor silvarum* (**A**: DsCREBBP; **B**: DsKAT6B; **C**: DsKAT5).

**Additional file 3**: **Fig. S3.** Full western blotting images for evaluation of the levels of H3K9 acetylation in *Dermacentor silvarum* under different cold treatments (**A**, **B**, **C**: cold treatment for 3, 6, and 9 days, respectively).

**Additional file 4: Fig. S4.** Full western blotting images for evaluation of the relative protein expression of KAT5 in *Dermacentor silvarum* under different cold treatments (**A**, **B**, **C**: cold treatment for 3, 6, and 9 days, respectively).

## Data Availability

The sequences characterized in the present study were deposited in NCBI under accession number of QQ851104, QQ851105, and QQ851106. The other data and materials that support the findings of this study are available from the corresponding author upon reasonable request.
