# Supplementary figures and images for: Molecular characterization and modulated expression of histone acetyltransferases during cold response of the tick Dermacentor silvarum (Acari: Ixodidae)
Source: Parasit Vectors. 2023 Oct 10;16:358. doi: 10.1186/s13071-023-05955-2 (PMC10566034; doi:10.1186/s13071-023-05955-2)

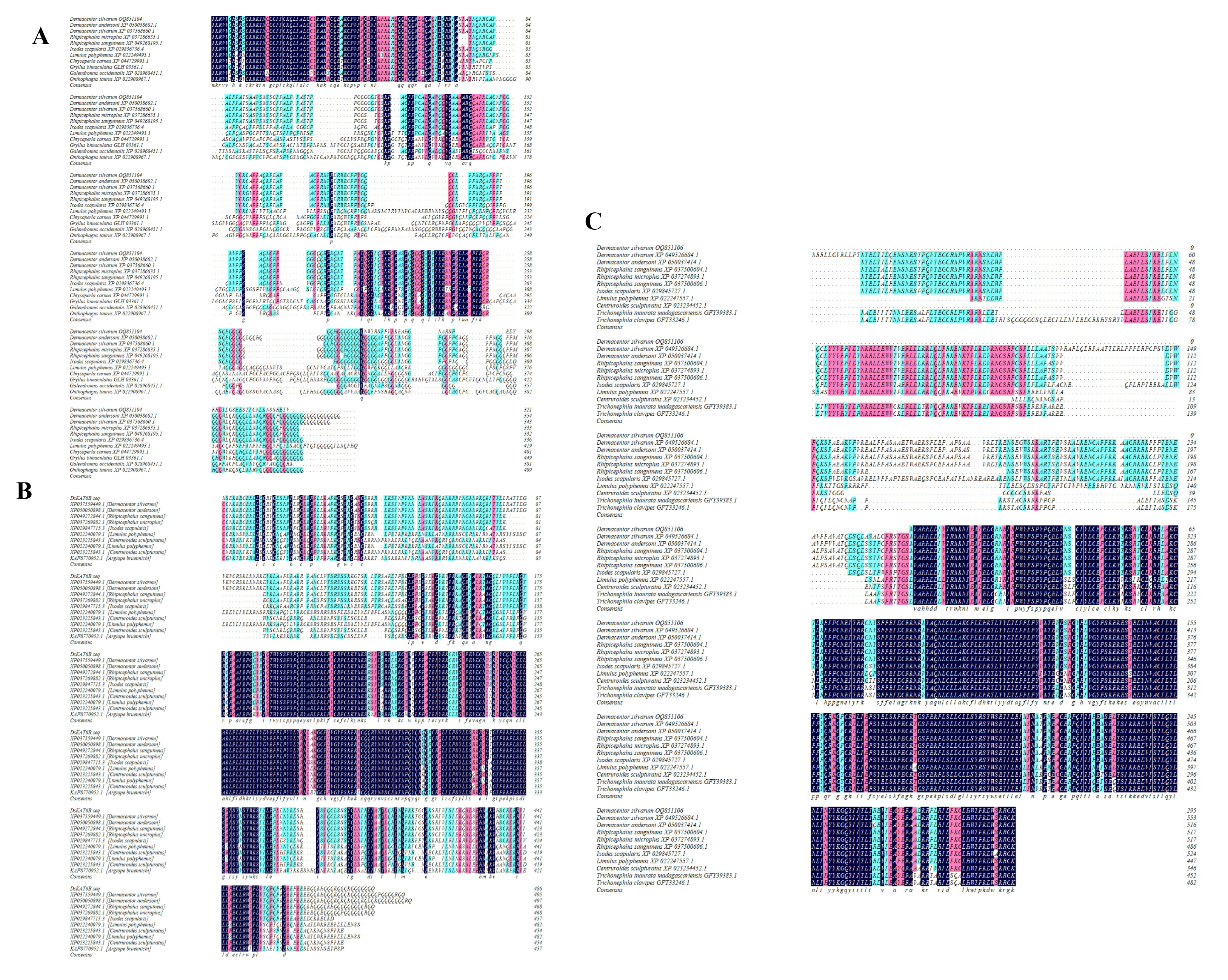

Supplement: Supplementary file 1 — Additional file 1: Fig. S1. Homology comparison of the histone acetyltransferases in Dermacentor silvarum (A: DsCREBBP; B: DsKAT6B; C: DsKAT5). [file 13071_2023_5955_MOESM1_ESM.tif]

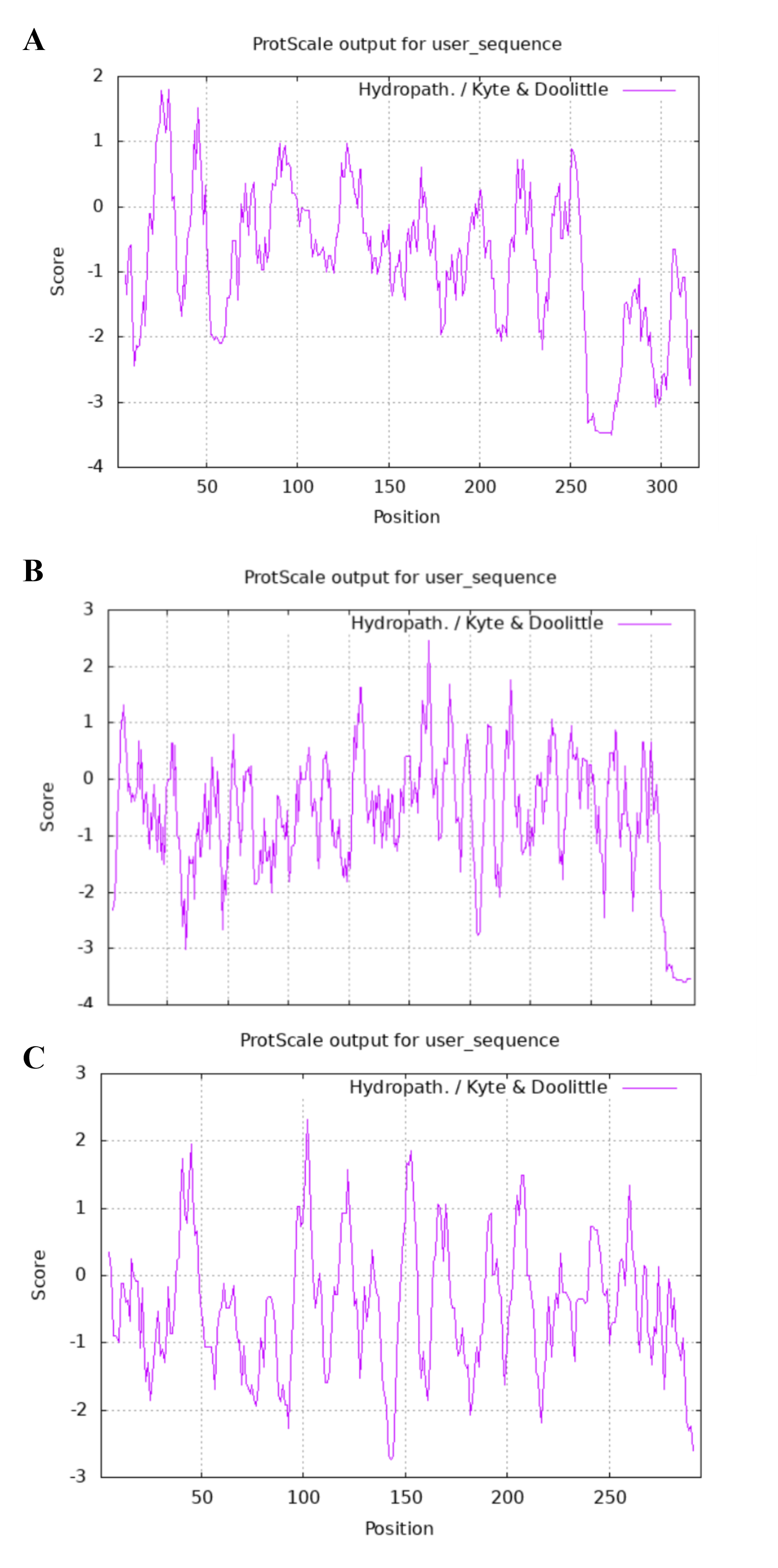

Supplement: Supplementary file 2 — Additional file 2: Fig. S2. Prediction of hydrophobicity of histone acetyltransferases in Dermacentor silvarum (A: DsCREBBP; B: DsKAT6B; C: DsKAT5). [file 13071_2023_5955_MOESM2_ESM.tif]

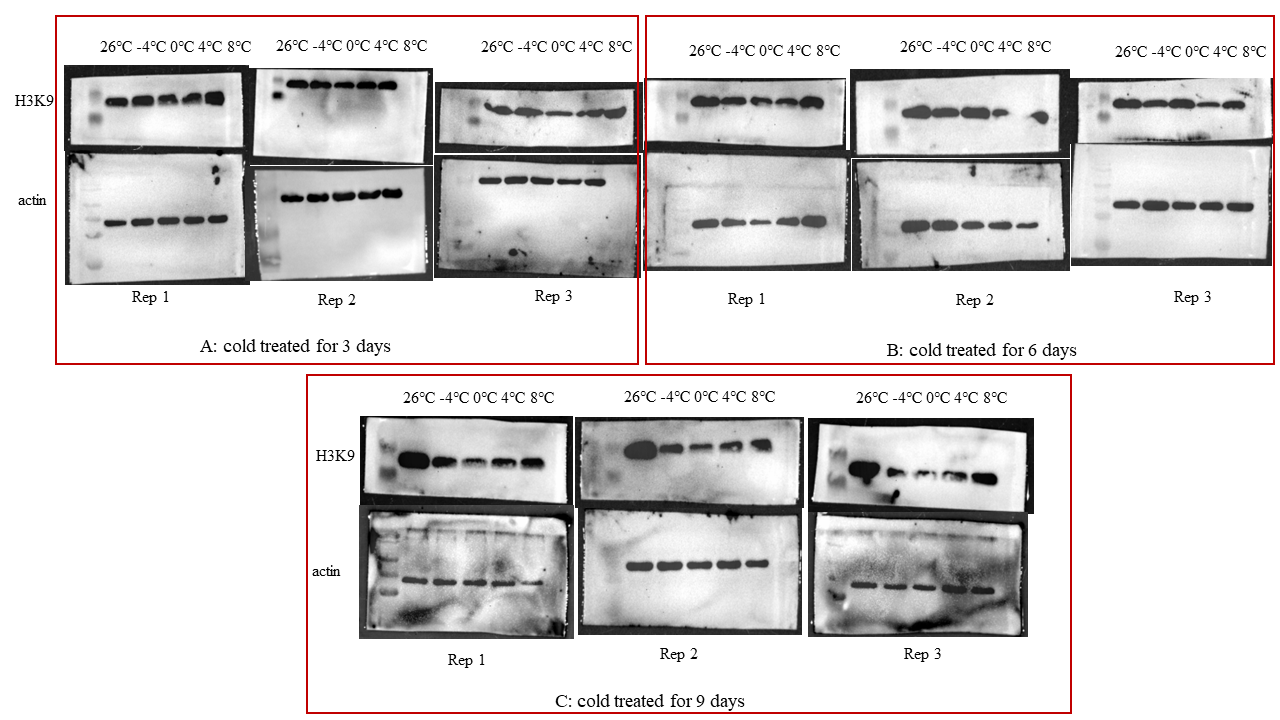

Supplement: Supplementary file 3 — Additional file 3: Fig. S3. Full western blotting images for evaluation of the levels of H3K9 acetylation in Dermacentor silvarum under different cold treatments (A, B, C: cold treatment for 3, 6, and 9 days, respectively). [file 13071_2023_5955_MOESM3_ESM.tif]

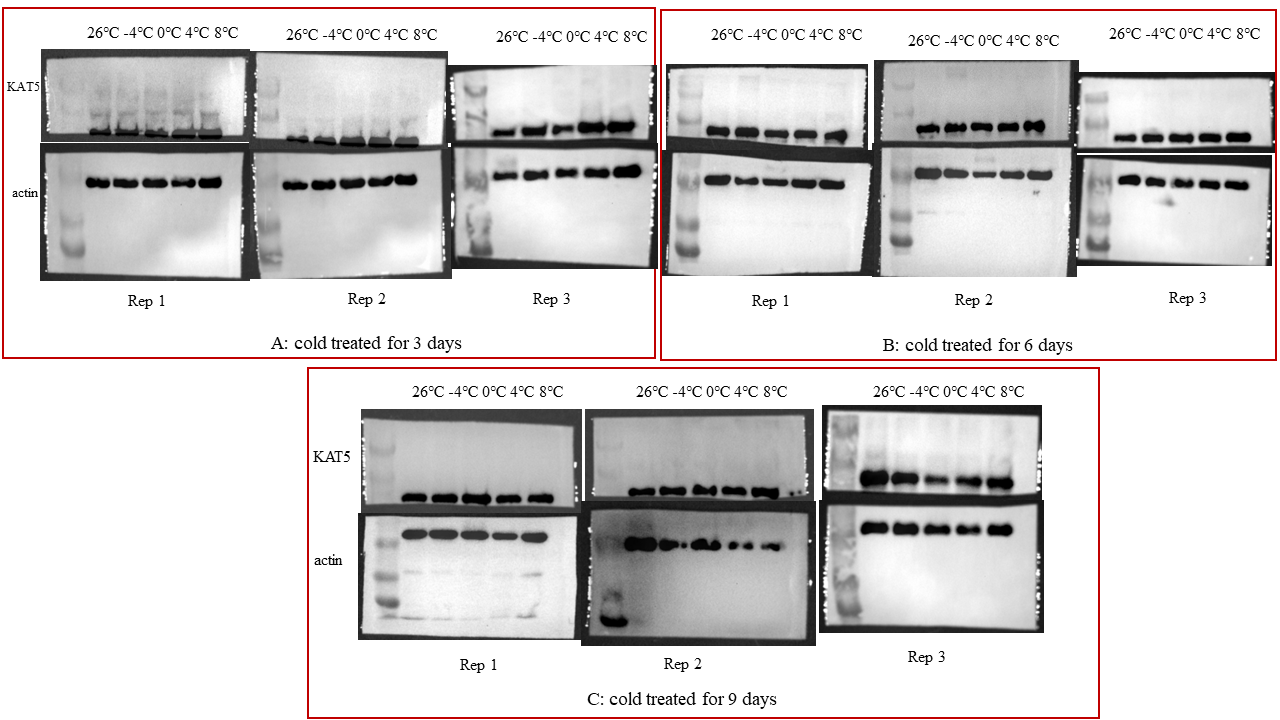

Supplement: Supplementary file 4 — Additional file 4: Fig. S4. Full western blotting images for evaluation of the relative protein expression of KAT5 in Dermacentor silvarum under different cold treatments (A, B, C: cold treatment for 3, 6, and 9 days, respectively). [file 13071_2023_5955_MOESM4_ESM.tif]
